# Moderating effect of self-esteem between perfectionism and avoidant restrictive food intake disorder among Lebanese adults

**DOI:** 10.1186/s12888-024-05762-8

**Published:** 2024-04-26

**Authors:** Roni Chaaya, Rabih Hallit, Diana Malaeb, Fouad Sakr, Mariam Dabbous, Sami El Khatib, Feten Fekih-Romdhane, Souheil Hallit, Sahar Obeid

**Affiliations:** 1 School of Arts and Sciences, Social and Education Sciences Department, Lebanese American University, Jbeil, Lebanon; 2 School of Medicine and Medical Sciences, Holy Spirit University of Kaslik, Jounieh, P.O. Box 446, Lebanon; 3 Department of Infectious Disease, Bellevue Medical Center, Mansourieh, Lebanon; 4 Department of Infectious Disease, Notre Dame des Secours University Hospital, Postal code 3, Byblos, Lebanon; 5 College of Pharmacy, Gulf Medical University, Ajman, United Arab Emirates; 6 School of Pharmacy, Lebanese International University, Beirut, Lebanon; 7 Department of Biomedical Sciences, School of Arts and Sciences, Lebanese International University, Bekaa, Lebanon; 8 Center for Applied Mathematics and Bioinformatics (CAMB), Gulf University for Science and Technology (GUST), Hawally, Kuwait; 9 The Tunisian Center of Early Intervention in Psychosis, Department of Psychiatry “Ibn Omrane”, Razi hospital, 2010 Manouba, Tunisia; 10 Faculty of Medicine of Tunis, Tunis El Manar University, Tunis, Tunisia; 11 Applied Science Research Center, Applied Science Private University, Amman, Jordan

**Keywords:** ARFID, Restrictive eating, Restrained eating, Self-esteem, Perfectionism, Lebanon

## Abstract

**Background:**

Avoidant Restrictive Food Intake Disorder (ARFID) is a new diagnosis added to the DSM-5 characterized by pathological eating habits without body image disturbances. Previous findings demonstrated a general association between high levels of perfectionism and low levels of self-esteem in association with general eating disorders. However, research is scant when it comes to ARFID specifically. Subsequently, although self-esteem is seen to moderate the association between perfectionism and general eating disorders, this research study aims to explore the same moderation but with ARFID specifically.

**Methods:**

For this study, 515 Lebanese adults from the general Lebanese population were recruited from all over Lebanon, 60.1% of which were females. The Arabic version of the Big Three Perfectionism Scale– Short Form (BTPS-SF) was used to measure self-critical, rigid and narcissistic perfectionism; the Avoidant/Restrictive Food Intake Disorder screen (NIAS) was used to score the ARFID variable; the Arabic-Single Item Self-Esteem (A-SISE) was the scale used to measure self-esteem.

**Results:**

Across the different perfectionism types, self-esteem was seen to moderate the association between narcissistic perfectionism and ARFID (Beta = − 0.22; *p* =.006). At low (Beta = 0.77; *p* <.001), moderate (Beta = 0.56; *p* <.001) and high (Beta = 0.36; *p* =.001) levels of self-esteem, higher narcissistic perfectionism was significantly associated with higher ARFID scores.

**Conclusion:**

This study brought to light some crucial clinical implications that highlight the need for interventions that help in the enhancement of self-esteem in patients with high perfectionism and ARFID. This study suggests that clinicians and healthcare professionals should focus more on risk factors influencing the development and maintenance of ARFID-like symptoms.

## Introduction

Avoidant Restrictive Food Intake Disorder (ARFID), a diagnosis first introduced in the fifth version of the Diagnostic and Statistical Manual of Mental Disorders (DSM-5), is distinguished by abnormal eating patterns without the body image disturbances characteristic of other eating disorders [1, 2]. More specifically, those diagnosed with ARFID fail to fulfill their nutritional requirements due to reasons unrelated to self-image worries, weight gain fears or medical or cultural concerns [3]. This disordered eating behavior can be associated with (1) being disinterested in food; (2) having a heightened visual, olfactory, or gustatory sensitivity to food; (3) exhibiting avoidance of potential consequences, such as choking that may arise from eating [3]. Unlike other eating disorders, ARFID is characterized by the absence of any fear related to weight gain [4]. Patients with ARFID are characterized by their inability to meet their nutritional requirements, with food avoidance being their primary characteristic [4]. Another distinction to make between ARFID on one hand, and anorexia nervosa and bulimia nervosa on the other hand, is the absence of body image disturbances [4]. Unlike other eating disorders, ARFID patients seek to improve their food intake habits and to gain weight; however, they find themselves unable to act upon this desire [4]. In addition, a distinction between ARFID patient and picky eaters should be made, where the former have clinically significant impairments in their eating habits, avoiding food to a degree where their energy is depleted, and their nutritional needs are not met [4]. Therefore, compared to anorexia and bulimia nervosa, patients with ARFID do not struggle with overevaluation of their weight nor do they have disturbances with their body image [2]. Individuals with ARFID commonly express apprehension regarding negative consequences of eating, such as the fear of choking or nausea, and being concerned about the immediate adverse effects of eating [5]. Moreover, while binge eating disorder is identified by frequent occurrences of uncontrollable overeating, on a weekly basis, accompanied with significant distress, ARFID is distinguished by limited food consumption, leading to deficiencies in nutrient-intake [2].

The diagnosis of ARFID can be established in people across all age groups whose restrictive eating patterns result in an insufficient nutritional intake, weight loss, an increased reliance on nutritional supplements, and an impairment in psychosocial functioning [6]. Additionally, medical abnormalities can ensue from ARFID such as abdominal pain and amenorrhea in females [3], as well as malnutrition and bradycardia [7]. Selective eating behaviors during childhood have been linked to the development of ARFID; however, limited research is available regarding the longitudinal trajectory of ARFID and its ultimate outcome [7]. Furthermore, anxiety disorders, autism spectrum disorders, and attention deficit and hyperactivity disorders have been shown to be comorbid with ARFID [7]. Depression, suicidal ideations, self-harm, and weight loss also correlated with symptoms of ARFID [8].

With ARFID being a disorder that is associated with both physical health complications as well as concurrent psychiatric conditions, recent findings have emerged regarding its treatment [9, 10]. Primarily, Cognitive Behavioral Therapeutic approach specialized for the treatment of children, adolescents, and adults with ARFID (CBT-AR) was developed [9, 10]. CBT-AR spans 20 to 30 sessions across four stages. The first stage consists of psycho-educating the patient about ARFID and allowing them to self-monitor their eating habits [9, 10]. The second stage involves planning the treatment, explaining nutritional deficiencies, identifying new foods that the patient can try to counteract these deficiencies while promoting weight gain and improving overall wellbeing [9, 10]. In stage 3, the therapist addresses the patient’s maintaining mechanisms while practicing exposure during and in-between sessions [9, 10]. Finally, in stage 4, the patient’s progress is discussed, and a relapse prevention plan is created [9, 10]. In this context, in a study conducted on patients aged 10–17, CBT-AR was reported to have notable enhancements in ARFID symptoms, including the introduction of new foods into the patients’ diets and significant weight gain [9]. While following the same 4-stage approach, another study implemented the CBT-AR among adults aged 18 to 55 years [10]. Concordant with the outcomes observed in children and adolescents, adults who underwent CBT-AR demonstrated substantial decrease in their ARFID symptoms [10]. This improvement was accompanied by notable weight gain and the incorporation of new foods into their diets [10]. Both mentioned studies proved the feasibility, acceptability and proof-of-concept of CBT-AR as being an efficacious treatment for children, adolescents [9] and adults [10] with ARFID.

On another hand, in children and adolescents, Family-Based Therapy (FBT) that is specialized to patients with ARFID, has been shown to be efficient through parental empowerment and psychoeducation on eating behaviors and nutrition [11]. In the latter case, exposure exercises were led by parents at home with the patient, leading to reduced stress [11]. A different study pointed out that, unlike other eating disorders, the treatment of ARFID should prioritize behavioral and nutritional strategies of treatment [4]. Additionally, the study also noted variations in treatment between short-term and long-term ARFID patients. Thus, short-term patients, having had healthy eating habits prior to the development of ARFID, can relate to a remembered standard of healthy eating through behavioral experiments and the help of a dietician [4]. On the contrary, therapy with long-term patients requires the minimization of certain symptoms rather than entirely removing the disorder [4]. Therefore, gaining more insight into the mechanisms underlying the development of ARFID might help advance knowledge on its prevention and intervention. In this study, we are focusing on the relationship between perfectionism and ARFID.

The development of ARFID symptoms has the most diverse etiology among all eating disorders, with a history of picky eating being one contributing factor [12]. Typical selective / picky eating is set apart from ARFID through the absence of substantial nutritional or psychosocial consequences [13], not always impacting weight status [6]. Yet, in severe cases of picky eating, the development of ARFID symptoms ensues, contributing to malnourishment, weight loss and psychosocial complications [6], with individuals rigidly rejecting any kind of novel foods, becoming more selective with age [12]. Individuals exhibiting selective eating behaviors have demonstrated cognitive and behavioral rigidities, including limited interests, perfectionism and challenges dealing with unfamiliar or new situations [14]. With selective eating behaviors being associated with ARFID and with cognitive and behavioral rigidity, case studies have shown that individuals with ARFID tend to exhibit this form of rigidity as well [14]. Perfectionism, which is a cognitive or behavioral contributor to the maintenance of psychopathology [15], has been seen in individuals exhibiting ARFID symptoms in the form of rigidity as to how food is prepared or presented or showing aversive responses to the sensory properties of food [14]. Concordantly, perfectionism was seen to be associated with both behavioral and cognitive food restraints [16].

It is important to highlight that perfectionism is a varied construct with different forms, one of which is described by the inclination to project one’s own perfectionistic standards onto others in a manner that is demanding and overly critical [17]. This other-oriented perfectionism is commonly referred to as ‘narcissistic perfectionism’ [17]. Researchers aimed to elucidate this phenomenon by developing a model illustrating that narcissistic perfectionism encompasses traits such as grandiosity, entitlement, high expectations from others and perfectionism directed towards others [17].

### Self-esteem as a moderator

As for self-esteem, it is synonymous with one’s personal evaluation of their own self-worth [18]. Low self-esteem manifests itself in a sense of hopelessness to change, affecting the adherence to therapy of patients with eating disorders. It also comes in the form of determination in achieving success in areas that matter to them, such as weight regulation and body shape; hence, making it more challenging to exhibit change in these specific areas [19]. In contrast, a study conducted in Saudi Arabia found no significant association between self-esteem and eating disorders [20]. On another hand, in patients with ARFID specifically, one study found that they tend to have higher self-esteem compared to patients with anorexia nervosa [21]. This can be attributed to the fact that ARFID patients struggle to find the desire to eat, rather than presenting body dissatisfaction or fear of gaining weight [4]. However, compared to those who eat freely, avoidant eaters tend to have lower levels of self-esteem [22]. Another study concluded that psychological distress, such as depressive symptoms and low self-esteem, acts as a risk factor that contributes to restrictive eating behaviors in adolescents, allowing for the maintenance and escalation of symptoms [23]. Studies have also demonstrated the moderating effect of low self-esteem between the negative affect associated with social rejection and restrictive eating behaviors, while high self-esteem acted as a protective factor moderating the relationship between the affective consequences of social rejection and restrictive eating [24].

As previously discussed, in patients with ARFID symptoms specifically, picky eating was shown to be associated with perfectionism [14]. Neophobia, which is defined as avoidance and unwillingness to try or eat new food [25], is a common characteristic seen in patients displaying symptoms of ARFID [13]. This neophobic tendency has been associated with lower self-esteem scores [26]. In elaboration, adult patients diagnosed with ARFID might feel the need to hide their eating patterns out of fear of being perceived as immature [27]. They may also refrain from eating in social settings to prevent drawing attention to their perfectionistic food preferences [27]. This avoidance behavior hinders their abilities to form new relationships, leading to social isolation and increased neophobia [27]. With social isolation being associated with low self-esteem [28] we can assume that the picky eating and neophobic characteristics of people with ARFID contribute to the low self-esteem in these individuals. It can also be assumed that it is this neophobic tendency, which is hindering these people’s ability to socialize, is reinforcing the perfectionistic selective eating behaviors seen in ARFID patients.

### The present study

To our knowledge, due to the scarce literature on ARFID in Lebanon, this would be the first study focusing on ARFID specifically in light of the two other variables among a sample of Lebanese participants. When discussing eating disorders, it is essential to comprehend the cultural factors that contribute towards their etiology [20], including in Arab countries and cultural background. In Saudi Arabia for instance, an Islamic nation, people adopt the teachings of the Holy Quran that advocates for restrictive eating behaviors [20]. In addition, food is perceived with high respect among Arab people, carrying a robust social, religious, and cultural significance [29]. Therefore, in an Arab country, where food is viewed highly, having ARFID can be perceived as impairing and challenging [29]. In a study conducted on a Saudi sample, the findings demonstrated the absence of a significant distinction in eating habits between Saudis who adopt an Arab-oriented lifestyle compared to those who are more Western-oriented [20]. Additionally, the study found no significant associations between eating disorders on one hand, and self-esteem and the use of social media on the other hand [20]. A different study showed a significant association between eating disturbances and increased exposure to Western media [30]. Therefore, the exploration of ARFID along with its associated characteristics and tendencies would aid in filling up the gaps in the literature, specifically within the Arab context.

More specifically, within the Lebanese context, a better understanding is needed regarding how perfectionism and self-esteem correlate with increased levels of avoidant and restrictive eating behaviors, and how self-esteem can moderate this relationship as well. In this study, our objective was to assess the moderating effect of self-esteem between perfectionism and ARFID symptoms [18]. Therefore, it is expected that self-esteem will moderate this association.

## Methods

### Study design

Using Google Forms, a questionnaire was developed and disseminated thought a variety of messaging platforms, such as WhatsApp, Instagram, and Facebook Messenger. Through a snowball sampling, 515 participants were recruited in this cross-sectional between February and March of 2023. To be eligible for participation, individuals needed to be Lebanese citizens residing in Lebanon and be 18 years old or older. Considering that an online survey was created for this study, internet access was a necessity, alongside a willingness to partake in the research study. Participants who declined to complete the questionnaire were excluded from the study. The assessment tools provided in the questionnaire were presented in a randomized order to mitigate any potential order-related biases. Participants were assured confidentiality and anonymity, while agreeing to complete the questionnaire voluntarily without any form of compensation. The average time required to fill out the questionnaire was around 20 min.

### Minimal sample size calculation

We used G*Power software to determine the sample size. The minimum required sample size was 457 participants, considering an alpha error of 5%, a power of 95%, a minimal model R-square of 5% (effect size of 5%) and allowing 9 predictors to be included in the model.

### Instruments

The questionnaire consisted of self-report scales. It also comprised of questions about sociodemographic details such as age, sex, education level, living area, marital status and Household Crowding Index (HCI), which is calculated by dividing the total count of people living in a household, excluding a newborn child, by the total number of rooms in that household, excluding the kitchen and bathrooms [31]. Participants self-reported their height and weight to compute their Body Mass Index (BMI) [32]. Three questions about physical activity intensity (1 = light activity to 5 = Heavy breathing and constant sweating), duration (1 = < 10 min to 4 = 30 or more minutes), and frequency (1 = less than once a month to 5 = daily or almost daily) were used to calculate a physical activity index by multiplying the three values (yielding a maximum score of 100). Higher scores reflect greater physical activity [33]. Regarding their perceived financial burden, respondents were asked to answer one question “How much pressure do you feel with regard to your personal financial situation in general?” on a scale from 1 to 10, with 10 referring to overwhelming pressure. The following measures have been used:

#### The big three perfectionism scale– short form (BTPS-SF)

The BTPS-SF, validated in Lebanon [34], is an abridged, 16-item scale of the 45-item BTPS, scored on a 7-point Likert scale from 1 (disagree strongly) to 7 (agree strongly) [35]. It includes three primary factors of perfectionism that tend to be overarching, namely rigid, self-critical, and narcissistic perfectionism [35]. Rigid perfectionism, characterized by the expectation of flawless performance from oneself [36], includes 4 items such as “I have a strong need to be perfect” [35]. The second factor, self-critical perfectionism, which is defined as having negative reactions to performance that is flawed and that others expect perfection from oneself [37], consists of 6 items such as “The idea of making a mistake frightens me” [35]. Narcissistic perfectionism, on the other hand, refers to the expectation of perfection from others in an overly critical and entitled manner [36], and consists of 6 items such as “I expect those close to me to be perfect” [35]. In this study, the internal reliability values were as follows: rigid perfectionism (*ω* = 0.85 / *α* = 0.85), self-critical perfectionism (*ω* = 0.86 / *α* = 0.86) and narcissistic perfectionism (*ω* = 0.83 / *α* = 0.83).

#### Avoidant/restrictive food intake disorder screen (NIAS)

Validated in Lebanon [29], the Arabic version of the NIAS was used to screen for ARFID symptoms [29]. This 9-item scale is scored on a 6-point Likert scale labeled from “strongly disagree” to “strongly agree”, with a total score ranging from 0 to 45, calculated by summing all 9 items [38]. It is composed of three factors– Picky eating, Appetite and Fear– each consisting of three items [29]. Subscale scores range from 0 to 15 and are calculated by summing their respective 3 items [38]. The higher the score on the NIAS, the more avoidant/restrictive the eating behavior is [39]. Cutoff values of ≥ 10, ≥ 9, and/or ≥ 10 have been proposed for capturing individuals who fit the NIAS dimensions: Picky eating, Appetite, and Fear respectively [39] (*ω* = 0.88 and *α* = 0.88 in this study).

#### The arabic-single item self-esteem (A-SISE)

The Arabic version of the Single Item Self-Esteem scale was used for this study, which consists of a 5-point Likert scale ranging from 1 (*not at all true to me*) to 5 (*very true of me*). The only item in this scale is “I have high self-esteem” [40]. The scale is validated in the Lebanese population and has been proven to be a valid and reliable instrument for the measurement of self-esteem [40].

### Statistical analysis

Data analysis was performed using SPSS software. The database contained no missing data. Reliability analysis of all scales and subscales was conducted by recording McDonald’s omega and Cronbach’s alpha values. The ARFID score was normally distributed (skewness and kurtosis values between ± 1) [41]. The Student t test was used to compare two means. Pearson’s correlation test was used to correlate two scores. The PROCESS Macro v3.4 model 1 (add-on for SPSS) [42] was used to conduct moderation models to examine the potential moderating effect of self-esteem (moderator) on the relationship between perfectionism (independent variable) and avoidant restrictive eating (dependent variable). Variables that demonstrated a *p* <.25 in the bivariate analysis were subsequently selected for inclusion in the moderation analysis as confounding factors. *P* <.05 denoted a statistically significant relationship.

## Results

### Sociodemographic and other characteristics of the sample

Five hundred fifteen adults participated in this study, with a mean age of 27.55 ± 10.92 years and 60.1% females. Other descriptive statistics of the sample can be found in Table 1. Furthermore, 17 (3.3%) had positive screen on any NIAS subscale (≥ 10 NIAS-picky eating, ≥ 9 NIAS-appetite, and ≥ 10 NIAS-fear).

**Table 1:** Sociodemographic and other characteristics of the sample (*N* = 515)

Variable	N (%)
Sex	
Male	155 (30.1%)
Female	360 (69.9%)
Marital status	
Single	377 (73.2%)
Married	138 (26.8%)
Education	
Secondary or less	84 (16.3%)
University	431 (83.7%)
Living area	
Urban	298 (57.9%)
Rural	217 (42.1%)
	**Mean ± SD**
Age (years)	27.55 ± 10.92
Household crowding index (persons/room)	1.15 ± 0.57
Body Mass Index (kg/m^2^)	24.27 ± 4.54
Physical activity	24.69 ± 19.79
Financial burden	5.33 ± 2.65
Self-esteem	3.49 ± 0.93
Avoidant restrictive eating	15.64 ± 8.48
Rigid perfectionism	12.30 ± 3.47
Self-critical perfectionism	17.23 ± 4.84
Narcissistic perfectionism	15.45 ± 4.49

### Bivariate analysis of factors associated with avoidant restrictive eating

The results of the bivariate analysis of factors associated with avoidant restrictive eating are summarized in Tables 2 and 3. The results showed that higher BMI (*r* = −.12) was significantly associated with lower avoidant restrictive eating, whereas higher rigid (*r* =.18), self-critical (*r* =.24) and narcissistic (*r* =.29) perfectionism were significantly associated with higher avoidant restrictive eating.

**Table 2:** Bivariate analysis of factors associated with avoidant restrictive eating

Variable	Mean ± SD	T	df	*P*
Sex		0.94	513	0.346
Male	16.17 ± 8.68			
Female	15.41 ± 8.40			
Marital status		− 0.56	513	0.573
Single	15.51 ± 8.53			
Married	15.99 ± 8.38			
Education		0.79	513	0.427
Secondary or less	16.31 ± 9.02			
University	15.51 ± 8.38			
Living area		-1.11	513	0.266
Urban	15.28 ± 8.10			
Rural	16.12 ± 8.97			

**Table 3:** Correlations of continuous variables with avoidant restrictive eating

	1	2	3	4	5	6	7	8	9	10
1. Avoidant restrictive eating	1									
2. Age	− 0.01	1								
3. Household crowding index	− 0.03	0.13**	1							
4. Financial burden	− 0.01	0.02	− 0.01	1						
5. Body Mass Index	− 0.12**	0.33***	− 0.09*	0.08	1					
6. Physical activity	− 0.001	− 0.11*	− 0.03	− 0.05	− 0.02	1				
7. Self-esteem	− 0.07	− 0.05	− 0.03	0.06	− 0.08	− 0.02	1			
8. Rigid perfectionism	0.18***	− 0.12**	− 0.09*	0.13**	− 0.13**	− 0.02	0.10*	1		
9. Self-critical perfectionism	0.24***	− 0.10*	0.001	0.10*	− 0.09*	− 0.05	− 0.08	0.60***	1	
10. Narcissistic perfectionism	0.29***	− 0.02	− 0.03	0.14**	− 0.01	− 0.04	0.02	0.55***	0.58***	1

*p <.05; **p <.01; ***p <.001

### Moderation analysis

The results of the moderation analysis are summarized in Table 4. Results were adjusted over body mass index. Higher perfectionism was significantly associated with higher avoidant restrictive eating in all three models. The interaction narcissistic perfectionism by self-esteem was significantly associated with ARFID scores (Beta = − 0.22; *p* =.006). At low (Beta = 0.77; *p* <.001), moderate (Beta = 0.56; *p* <.001) and high (Beta = 0.36; *p* =.001) levels of self-esteem, higher narcissistic perfectionism was significantly associated with higher ARFID scores (Table 5). The same results were found in the total sample, males and females separately.

**Table 4:** Moderation analysis taking each perfectionism subscale as the independent variable, self-esteem as the moderator and avoidant restrictive eating as the dependent variable

	Beta	t	*p*	95% CI	Beta	t	*p*	95% CI	Beta	t	*p*	95% CI
	Total sample				Males				Females			
**Model 1: Rigid perfectionism as the independent variable.**												
Rigid perfectionism	0.99	2.33	**0.020**	0.16; 1.82	1.99	2.51	**0.013**	0.42; 3.55	0.61	1.21	0.228	− 0.38; 1.60
Self-esteem	0.99	0.70	0.488	-1.80; 3.77	2.87	1.06	0.290	-2.47; 8.22	0.27	0.16	0.871	-3.01; 3.55
Interaction rigid perfectionism by self-esteem	− 0.15	-1.36	0.174	− 0.37; 0.07	− 0.37	-1.75	0.083	− 0.78; 0.05	− 0.07	− 0.54	0.587	− 0.33; 0.19
**Model 2: Self-critical perfectionism as the independent variable.**												
Self-critical perfectionism	0.67	2.36	**0.019**	0.11; 1.24	1.14	2.02	**0.045**	0.03; 2.24	0.52	1.57	0.116	− 0.13; 1.18
Self-esteem	0.77	0.56	0.573	-1.91; 3.45	1.49	0.56	0.576	-3.75; 6.72	0.56	0.35	0.723	-2.56; 3.69
Interaction self-critical perfectionism by self-esteem	− 0.08	-1.01	0.312	− 0.23; 0.07	− 0.15	− 0.97	0.334	− 0.45; 0.15	− 0.06	− 0.63	0.530	− 0.23; 0.12
**Model 3: Narcissistic perfectionism as the independent variable.**												
Narcissistic perfectionism	1.34	4.57	**< 0.001**	0.76; 1.91	1.84	3.19	**0.002**	0.70; 2.98	1.13	3.30	**0.001**	0.46; 1.80
Self-esteem	2.69	2.05	**0.041**	0.12; 5.27	3.89	1.50	0.135	-1.22; 9.00	2.25	1.46	0.144	− 0.77; 5.27
Interaction narcissistic perfectionism by self-esteem	− 0.22	-2.77	**0.006**	− 0.38; − 0.07*	− 0.32	-2.00	**0.047**	− 0.64; − 0.004*	− 0.18	-1.97	**0.049**	− 0.37; − 0.001*

*indicates significant moderation; results adjusted over the following variables: own a car and alcohol drinking. The LOG of the eating attitudes score was used for this analysis

**Table 5:** Conditional effects of the focal predictor (narcissistic perfectionism) at values of the moderator (self-esteem)

Moderator	Beta	t	*p*	95% CI	Beta	t	*p*	95% CI	Beta	t	*p*	95% CI
	Total sample				Males				Females			
Low (= 2.57)	0.77	7.00	**< 0.001**	0.55; 0.98	1.00	4.95	**< 0.001**	0.60; 1.40	0.66	5.03	**< 0.001**	0.40; 0.92
Moderate (= 3.49)	0.56	7.15	**< 0.001**	0.41; 0.72	0.71	4.86	**< 0.001**	0.42; 1.00	0.49	5.22	**< 0.001**	0.31; 0.67
High (= 4.42)	0.36	3.34	**0.001**	0.15; 0.57	0.42	1.98	**0.050**	0.001; 0.83	0.32	2.55	**0.011**	0.07; 0.56

Numbers in bold indicate significant *p* values

## Discussion

The aim of this study was primarily to explore the moderating role of self-esteem in the association between perfectionism and ARFID symptoms. It was seen that high perfectionism scores were associated with more avoidant restrictive eating, consistent with the findings of other studies [16, 43]. Through a moderation analysis of three different models, self-esteem was shown to moderate the effect of narcissistic perfectionism, but not self-critical and rigid perfectionism, on ARFID symptoms. In other words, higher levels of narcissistic perfectionism were significantly associated with higher levels of ARFID at low, moderate, and high self-esteem levels. With perfectionism often observed in individuals with ARFID symptoms manifesting as rigidity in food presentation [14], and considering narcissistic perfectionism as perfectionism directed towards others [17], we can explore the association between elevated levels of narcissistic perfectionism and ARFID. This association may stem from the notion that individuals with ARFID could demonstrate perfectionistic tendencies in how food is presented or prepared, alongside exhibiting aversive responses to the sensory attributes of food. Thus, based on the results of this study, it can be concluded that both self-esteem and perfectionism are related to ARFID, in agreement with the findings of other studies that showed a positive association between self-esteem and restrained eating [44] and between perfectionism and restrictive eating patterns [43, 45].

Previous findings illustrated that low self-esteem [21, 46] and high perfectionism [21] were characteristic of Anorexia Nervosa rather than ARFID in children and adolescents. This can be highlighted by the fact that people with ARFID have more long-term symptoms compared to those with Anorexia, leading the former to habituate to their symptoms over time [46]. The current study, however, demonstrated that these two variables, low self-esteem, and high perfectionism, are factors contributing to high ARFID scores in adults. The high levels of perfectionism seen in this study in individuals with ARFID can be explained by the rigidity exhibited by those who display selective eating behaviors, considering that perfectionism acts as a cognitive and behavioral risk factor contributing to the maintenance of ARFID symptoms [14, 15]. On another hand, the low self-esteem seen in individuals displaying ARFID symptoms in this study can be attributed to the association that exists between neophobia, a common tendency seen in ARFID patients [13], and low self-esteem [25].

In this study, higher levels of rigid, self-critical, and narcissistic perfectionism were seen to be associated with higher avoidant restrictive eating. Even in the presence of high levels of self-esteem, perfectionism still correlated with high NIAS scores. However, lower perfectionism scores are commonly witnessed in ARFID patients compared to those with anorexia or bulimia nervosa [47]. Thus, it is important to imply that perfectionism differs based on the profile of the eating disorder being studied and context-dependent on the eating disorder [47]. Behavioral restrictions in eating have been related to greater levels of self-critical perfectionism. This is seen as problematic considering the physical consequences of these behavioral restrictions [16]. Additionally, self-critical perfectionism was seen to negatively relate to improvements in therapy [48]. Self-critical perfectionism has also been negatively correlated with mindfulness eating, adding proof to why someone with high self-critical perfectionism might engage in restrictive eating behaviors [49]. Both self-critical and rigid perfectionism have been associated with a greater likelihood of developing eating disorders, and a rise in mental illnesses [50]. In a different study, rigid perfectionism was found to be a risk factor for the development of eating disorders in general [51]. On another hand, narcissistic perfectionism has not been seen to be associated with eating attitudes in a different study [50]. Despite that research about the association between perfectionism and ARFID is scant, it can be deduced that the former is related to higher disordered eating generally, and restrictive eating behaviors specifically [16]. Therefore, future research should focus on mitigating the symptoms of ARFID through interventions that target both perfectionism and self-esteem, as well as studying the role of body dissatisfaction in the triadic model explored in this study between the three variables.

Furthermore, in the present study, self-esteem was found to be a moderator between perfectionism and restrictive eating behaviors. Previous studies have shown that self-esteem plays a moderating role in the relationship between body dissatisfaction (which is linked to perfectionism) and restrained eating [44]. Although body dissatisfaction is not a characteristic of ARFID [4], the moderating effect of self-esteem was witnessed between perfectionism and restrained eating in this study. Higher levels of self-esteem [21, 46] and lower levels of perfectionism [21] were seen in patients with ARFID compared to those with Anorexia Nervosa, which can suggest that high levels of self-esteem contributes to minimizing the impact of perfectionism on the development of ARFID symptoms compared to low levels.

### Limitations

The male-to-female ratio in this study was disproportionate, which might have given rise to biases in the results of the study. Another limitation is the derivation of the results from self-report scales, giving rise to the possibility of social desirability affecting the scores and influencing the outcome of the study [18]. This study consisted of a non-clinical sample of participants [16]. The snowball technique method used to recruit participants predisposes us to a selection bias; therefore, our sample does not represent the general Lebanese population. Finally, this study used the perfectionism scale that is limited to three subscales; other perfectionism dimensions also exist, such as personal standards, concern over mistakes, parental expectations, doubting of actions, and organization [52]. Future research can also explore different perfectionism dimensions other than the three used in this study which can also provide a more detailed, comprehensive understanding of their relationship with ARFID.

## Conclusion

This study adds to the missing literature on the triadic model delineating the moderating role of self-esteem between perfectionism and ARFID. Additionally, this study brought to light some crucial clinical implications that highlight the need for interventions, which would help enhance self-esteem in patients with high perfectionism and ARFID. This study suggests that clinicians and healthcare professionals should focus more on risk factors influencing the development and maintenance of ARFID-like symptoms. Based on the results of this study, by implementing interventions that work on improving people’s self-esteem or the management of perfectionistic tendencies, symptoms of ARFID are likely to be reduced. Additionally, awareness sessions should be implemented within the Lebanese context to highlight the risk factors that contribute to ARFID and provide tips on how to manage these symptoms to enhance one’s eating habits. Therefore, it is crucial to improve the self-esteem of people exhibiting ARFID symptoms and those reporting high levels of perfectionism. An experimental approach to this study would also be beneficial by integrating a clinical ARFID sample alongside a healthy control group which can provide a deeper comprehension of the studied associations; thus, enhancing the validity of the findings in clinical settings.

## Data Availability

All data generated or analyzed during this study are not publicly available due the restrictions from the ethics committee, but are available upon a reasonable request from the corresponding author.
